# Transcutaneous Vagus Nerve Stimulation (tVNS) Improves High-Confidence Recognition Memory but Not Emotional Word Processing

**DOI:** 10.3389/fpsyg.2020.01276

**Published:** 2020-07-09

**Authors:** Manon Giraudier, Carlos Ventura-Bort, Mathias Weymar

**Affiliations:** Department of Biological Psychology and Affective Science, University of Potsdam, Potsdam, Germany

**Keywords:** transcutaneous vagus nerve stimulation, salivary alpha-amylase, emotion, words, episodic memory, recognition, recollection, confidence

## Abstract

Previous clinical research found that invasive vagus nerve stimulation (VNS) enhanced word recognition memory in epileptic patients, an effect assumed to be related to the activation of brainstem arousal systems. In this study, we applied non-invasive transcutaneous auricular VNS (tVNS) to replicate and extend the previous work. Using a single-blind, randomized, between-subject design, 60 healthy volunteers received active or sham stimulation during a lexical decision task, in which emotional and neutral stimuli were classified as words or non-words. In a subsequent recognition memory task (1 day after stimulation), participants' memory performance on these words and their subjective memory confidence were tested. Salivary alpha-amylase (sAA) levels, a putative indirect measure of central noradrenergic activation, were also measured before and after stimulation. During encoding, pleasant words were more accurately detected than neutral and unpleasant words. However, no tVNS effects were observed on task performance or on overall sAA level changes. tVNS also did not modulate overall recognition memory, which was particularly enhanced for pleasant emotional words. However, when hit rates were split based on confidence ratings reflecting familiarity- and recollection-based memory, higher recollection-based memory performance (irrespective of emotional category) was observed during active stimulation than during sham stimulation. To summarize, we replicated prior findings of enhanced processing and memory for emotional (pleasant) words. Whereas tVNS showed no effects on word processing, subtle effects on recollection-based memory performance emerged, which may indicate that tVNS facilitates hippocampus-mediated consolidation processes.

## 1. Introduction

One of the core aspects of adaptive behavior is the detection and remembrance of emotionally salient information. Ample evidence suggests that emotional content impacts various stages of processing, from initial encoding to later long-term retrieval (Dolcos et al., 2017, 2020b). For instance, emotionally salient events capture more attentional resources and are detected more efficiently than their neutral counterparts (Dolan, 2002). This effect has been demonstrated across a range of paradigms, including visual search (Fox et al., 2000), spatial cueing (Armony and Dolan, 2002), dot-probe (Mogg et al., 1997), and passive viewing tasks (Schupp et al., 2006), and using a variety of stimuli, such as emotional facial expressions (Fox et al., 2000; Schupp et al., 2004), images (Öhman et al., 2001; Armony and Dolan, 2002; Weymar et al., 2011), and non-verbal vocalizations (Sauter and Eimer, 2010). Similarly, for lexical material, an overall advantage for emotional (pleasant and unpleasant) words is consistently reported in lexical decision and reading tasks (Eviatar and Zaidel, 1991; Ortigue et al., 2004; Kanske and Kotz, 2007; Herbert et al., 2008; Schacht and Sommer, 2009; Scott et al., 2009; for a review see Kissler et al., 2009). These emotional benefits in attention may further influence memory formation and consolidation (Dolcos and Cabeza, 2002; Kensinger, 2009; Lang and Bradley, 2010), particularly during sleep (Wagner et al., 2006; Payne et al., 2008; Nishida et al., 2009), leading to increased memory retrieval for emotionally relevant compared with neutral information (Bradley et al., 1992; Weymar and Hamm, 2013; Dolcos et al., 2020b). Previous studies demonstrated a memory advantage for emotional images (Hamann et al., 1999), even 1 year after encoding, as evidenced by higher recall rates (Bradley et al., 1992) and recognition rates (Dolcos et al., 2005; Weymar et al., 2011), but also better memory for other material, including stories (Cahill and McGaugh, 1995), faces (Righi et al., 2012), and words (Phelps et al., 1997; Doerksen and Shimamura, 2001; Kensinger and Corkin, 2003). In addition to enhanced memory accuracy, emotional events are also retrieved more vividly, with more subjective confidence, and with enhanced recollective experience (i.e., recollection, the conscious retrieval of specific contextual details of the encoding episode) than for neutral events (Ochsner, 2000; Sharot et al., 2004; Dolcos et al., 2005; Weymar et al., 2009, 2010; Rimmele et al., 2012), which are often remembered with less confidence and less contextual information (i.e., familiarity) (D'Argembeau and Van der Linden, 2004; Sharot et al., 2007). A particular characteristic of the mnemonic advantage for emotionally relevant material is that it seems to be particularly sensitive to arousal (exciting vs. calming) rather than valence (pleasant vs. unpleasant), leading to prioritized perception and memory of emotionally (pleasant and unpleasant) arousing information at the expense of neutral, less-relevant information (Mather and Sutherland, 2011; Mather et al., 2016).

An influential neuroscientific theory (McGaugh, 2015) derived from animal and human studies suggests that better memory for unpleasant and pleasant events is related to the interaction between emotion-specific regions (e.g., amygdala) and memory-related regions (e.g., hippocampus) and is mediated by the afferent influence of stress hormones (epinephrine and glucocorticoids from adrenal glands) released during and after emotionally arousing experiences (Cahill and McGaugh, 1998; McIntyre et al., 2012; McGaugh, 2015). Critically, one of the pathways by means of which the stress hormones project to the amygdala consists of the afferent fibers of the vagus nerve (McIntyre et al., 2012). Specifically, the release of epinephrine in the adrenal gland modulates the activity of the vagus nerve, which consequently exerts influence on the locus coeruleus (LC) via the nucleus of the solitary tract (Van Bockstaele et al., 1999; McIntyre et al., 2012). The LC is the main source of noradrenergic neurons in the brain, and its activation favors the release of norepinephrine (NE) in a variety of cortical and subcortical brain areas, including the amygdala and hippocampus. In turn, activity of the LC may facilitate the formation, consolidation, and retrieval of emotional memories (Sterpenich et al., 2006; Groch et al., 2011; McIntyre et al., 2012; Clewett et al., 2018). Giving support to this assumption, prior research in animals and humans using implanted vagus nerve stimulation (VNS) found that invasive vagus nerve activation enhances long-term memory for inhibitory avoidance in rats (Clark et al., 1998) and modulates word recognition memory in humans (Clark et al., 1999), an effect assumed to be modulated by the LC-NE system (Hassert et al., 2004).

Recently, transcutaneous vagus nerve stimulation (tVNS) has been introduced as a novel brain stimulation tool that can activate the vagus nerve—in a non-invasive fashion—via the auricular branch (Van Leusden et al., 2015). Indeed, recent brain imaging studies have shown that tVNS modulates activity in the LC and areas innervated by this region, including the insula, amygdala, hippocampus, and thalamus (Dietrich et al., 2008; Kraus et al., 2013; Yakunina et al., 2017); tVNS also increased P300b amplitudes (Rufener et al., 2018; Ventura-Bort et al., 2018; Lewine et al., 2019; but see also Warren et al., 2019), an attention-related event-related potential (ERP) component putatively associated with phasic activity of the LC-NE system (Nieuwenhuis et al., 2005), and salivary alpha-amylase (sAA) levels (Ventura-Bort et al., 2018; Warren et al., 2019; but see also Koenig et al., 2019), an indirect marker of endogenous noradrenergic activation in the brain (Chatterton et al., 1996; Warren et al., 2017). Additionally, tVNS was found to modulate a variety of other cognitive and affective processes, such as cognitive control (Sellaro et al., 2015, 2018; Steenbergen et al., 2015; Fischer et al., 2018; Keute et al., 2019), associative memory (Jacobs et al., 2015), fear extinction (Burger et al., 2016; Szeska et al., 2020), and emotion recognition (Colzato et al., 2017), which may be related to the activation of LC-mediated NE following tVNS.

In the current study, we continued this line of research on the impact of non-invasive vagal stimulation on cognitive and affective functions by directly focusing on its effect on emotional encoding and memory. To extend the work of Clark et al. (1999), who found enhanced word recognition memory following VNS, we used emotional and neutral words as stimulus material. In a single-blind, randomized, between-subject design, healthy participants received either tVNS or sham stimulation while performing a lexical decision task with words and non-words. As in prior studies (Ventura-Bort et al., 2018; Warren et al., 2019), we measured sAA levels before and after stimulation to assess changes in noradrenergic activation following tVNS. One day later, participants performed a surprise recognition memory task in which previously encoded words and new words were presented. In the lexical decision task, we expected to replicate the advantage of lexical access for emotionally relevant information (e.g., Schacht and Sommer, 2009; Scott et al., 2009) as indicated by enhanced accuracy rates and reduced response times for emotional (unpleasant and pleasant) words than for neutral words. Given that emotional words undergo prioritized processing over neutral words as indicated by previous ERP studies (e.g., Schacht and Sommer, 2009), and assuming that tVNS increases the arousal level, which may enhance perception and memory for salient emotional information (at the expense of less-relevant neutral information) (Mather and Sutherland, 2011; Mather et al., 2016), we expected that tVNS would lead to higher rates of accuracy and reduced response times particularly for emotional words. In the recognition memory task, we predicted enhanced recognition performance for emotional compared to neutral words, reflected in greater discrimination accuracy and shorter response times. Because emotional stimuli are often associated with (hippocampus-driven) recollection-based memory processes (e.g., Doerksen and Shimamura, 2001 and Kensinger and Corkin, 2003 for words, and Ochsner, 2000, Dolcos et al., 2005, Weymar et al., 2009, Weymar et al., 2010, and Dolcos et al., 2020a for scenes), we speculated that tVNS, compared to sham stimulation, would particularly increase memory for emotional words that were retrieved with high subjective confidence (Yonelinas, 2001; Wixted and Stretch, 2004; Weymar et al., 2009). As a marker of endogenous noradrenergic activation, increased sAA levels were expected after tVNS compared to sham stimulation.

## 2. Materials and Methods

### 2.1. Participants

Sixty-one healthy psychology students (47 female; *M*_age_ = 23.39 years, SD_age_ = 4.67 years) from the University of Potsdam participated in the study for course credits. All participants provided informed written consent for the experimental protocol, which was approved by the ethics committee of the University of Potsdam and in accordance with the Declaration of Helsinki. All participants were native German speakers with normal or corrected-to-normal vision (54 right-handed). Prior to the first session, participants were phone-screened and invited to participate upon passing the following exclusion criteria: neurological or psychiatric disorders, brain surgery, use of medication or drugs, pregnancy, history of migraine or epilepsy, cardiac diseases, metal pieces in the body (e.g., pacemaker), and active implants or physical alterations in the ear (e.g., cochlear implant). One participant was excluded from the analyses for medical reasons, leaving a final sample of sixty participants (46 female; *M*_age_ = 23.45 years, SD_age_ = 4.87 years; 53 right-handed).

### 2.2. Procedure

#### 2.2.1. Stimulus Materials

Overall, 400 German words were selected from the Berlin Affective Word List Reloaded (BAWL-R) database (Võ et al., 2009). Based on their normative ratings, which ranged from −3 (very unpleasant; e.g. Grab/coffin) to 3 (very pleasant; e.g. Geschenk/gift) for valence and from 1 (low arousal; e.g. Eimer/bucket) to 5 (high arousal; e.g. Irrsinn/insanity) for arousal, we selected three different categories (*M*_valence_ = −1.50, SD_valence_ = 0.55, *M*_arousal_ = 3.59, SD_arousal_ = 0.38), neutral (*M*_valence_ = 0.11, SD_valence_ = 0.29, *M*_arousal_ = 2.23, SD_arousal_ = 0.29), and pleasant (*M*_valence_ = 1.32, SD_valence_ = 0.55, *M*_arousal_ = 3.33, SD_arousal_ = 0.27). The collection of all 400 words was divided into two sets of 200 stimuli each. The sets were matched on the basis of hedonic valence, arousal, and various lexical and sublexical variables, including word imageability, word length (numbers of letters, phonemes, syllables), word frequency, and orthographic neighborhood density (*ps* > 0.13). One set of words (100 emotional and 100 neutral) was presented in a lexical decision task and served as the basis for generating 200 non-words by randomly substituting one letter at a random position in each of the given words (e.g., *Eimer* → *Eimej*; *Grab* → *Grkb*). The same set of old words was presented in an explicit recognition memory task together with the set of 200 new words.

#### 2.2.2. Encoding Session: Lexical Decision Task

**Figure 1:**
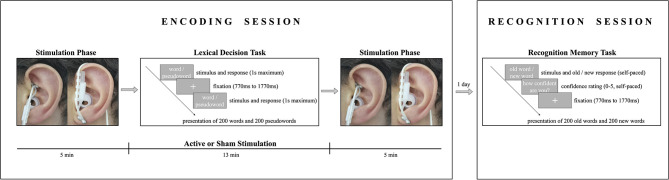
Schema illustrating the encoding and recognition procedures. In the encoding session, participants received either active stimulation to the cymba conchae **(left)** or sham stimulation to the earlobe **(right)** before, during, and after a lexical decision task. In the lexical decision task, participants indicated if the presented string was a German word by pressing a response button, or if it was a non-word by not responding. In the recognition session one day after encoding, participants indicated whether or not the presented string was a word that they had seen during the lexical decision task and were asked to indicate their recognition confidence on a scale from 0 (not confident) to 5 (absolutely confident).

The study consisted of two experimental sessions (lexical decision task and recognition memory task), which took place on two consecutive days (see Figure 1). During encoding, participants received either active stimulation or sham stimulation for 23 min while performing a lexical decision task in a sound-attenuated, dimly lit room. Subjects were familiarized with the experimental protocols (though no mention of a memory task was made) and randomly assigned to one of the two experimental groups (active stimulation or sham stimulation). Before undergoing stimulation, participants' heart rate, blood pressure (systolic and diastolic), and sAA levels were measured. Subjects were familiarized with the stimulator device, and an individual stimulation intensity was determined for each participant to ensure a maximum strong stimulation without pain (for the procedure see Ventura-Bort et al., 2018). Participants were stimulated before (5 min), during (13 min), and after (5 min) the lexical decision task.

Each trial began with a fixation cross presented in the middle of the screen for an interval that varied randomly between 770 and 1,770 ms. Thereafter, a letter string (i.e., a word or non-word) was presented until a response was given or until a time-out of 1 s. Participants were asked to indicate as quickly and accurately as possible if the presented string was a German word by pressing a response button with their index finger of the dominant hand; if a non-word was presented, participants were instructed not to respond. The strings were presented in two blocks of 200 trials each in a pseudorandomized order to control for confounding effects of word order. The order of blocks was randomized across participants, and the sequence of trials in each block was pseudorandomized under the constraints that words with identical arousal levels and valence levels were not presented more than two times in a row. The same word type (word or non-word) was not presented more than three times in a row. After stimulation, participants' sAA levels, heart rate, and blood pressure were measured a second time and they completed a tVNS adverse effects questionnaire in which they had to indicate, on a 7-point scale (1 being *not at all* and 7 being *very much*), how much they had experienced headache, nausea, dizziness, neck pain, muscle contractions in the neck, stinging sensations under the electrodes, skin irritation in the ear, fluctuations in mental concentration or feelings, and other unpleasant feelings or adverse effects.

#### 2.2.3. Recognition Session: Explicit Recognition Memory Task

One day after encoding, participants performed an old/new recognition task with self-paced response and a rating-based recognition confidence task on a 6-point scale (see Figure 1). Each trial began with a fixation cross presented in the middle of the screen for an interval that varied randomly between 770 and 1,770 ms, followed by a string that was presented until a response was given. The words were presented in two blocks of 200 trials each in a pseudorandomized order to control for confounding effects of word order. Participants were presented one word at a time and were asked to decide whether they had previously seen the word during encoding (old word) or not (new word) by pressing the corresponding response button on a keyboard. Hand assignment for the response buttons for old and new words was counterbalanced across participants. Following the old/new judgment, participants were asked to rate their confidence in memory by pressing the corresponding key in a Likert scale ranging from 0 (not confident) to 5 (absolutely confident) in order to assess the contribution of recollection and familiarity-based memory.

### 2.3. Transcutaneous Vagus Nerve Stimulation

In the current study, a single-blind, active stimulation-sham stimulation, randomized between-subject design was employed. Transcutaneous VNS was applied by stimulating the auricular branch of the vagus nerve using a recently engineered and non-invasive device (Cerbomed GmbH, Erlangen, Germany). The tVNS stimulator consists of two electrodes connected to a wired neurostimulating device; for the active stimulation it was placed in the left cymba conchae, an area innervated by the auricular branch of the vagus nerve, and for the sham stimulation it was placed on the left earlobe, an area that has been found to be free of cutaneous vagal innervation (Peuker and Filler, 2002; Ellrich et al., 2011) (see Figure 1). Stimulation alternated between on and off phases every 30 s and was delivered with a pulse width of 200–300 ms at 25 Hz. The stimulation intensity was determined individually for each participant by applying increasing and decreasing sequences of 10 s stimulation trials. Participants were asked to give direct feedback on how they perceived each stimulation intensity on a 10-point scale ranging from no perception (1) and light tingling (3) to strong tingling (6) and pain (10). The increasing sequence started from an intensity of 0.1 mA and increased stepwise in 0.1 mA increments until the subject reported a slight feeling of pain (corresponding to a subjective sensation of 9 on the scale). Before starting the decreasing series, the same intensity was repeated and then decreased stepwise in 0.1 mA increments until a subjective sensation of 6 or below was experienced (cf. Ventura-Bort et al., 2018). This protocol was repeated twice and the average of the intensities rated as 8 was used as the stimulation threshold. The individual stimulation intensities varied from 0.5 to 2.5 mA for the sham (earlobe) stimulation group (*M*_sham_ = 1.31, SD_sham_ = 0.50) and from 0.5 to 3.5 mA for the active (cymba conchae) stimulation group (*M*_active_ = 1.48, SD_active_ = 0.59). Stimulation intensities did not differ significantly between the groups [*t*(1, 58) = 1.08, *p* = 0.28, *d* = 0.28].

### 2.4. Autonomic Measures

To investigate the effects of tVNS on autonomic reactivity, subjects' heart rate and blood pressure (diastolic and systolic) were measured using the Intelli Wrap Manschette M500 device (Omron Healthcare, Medizintechnik Handelsgesellschaft mbH, Mannheim, Germany). In addition, their sAA levels were measured as a potential marker of endogenous noradrenergic activity (Warren et al., 2017). This was done by collecting saliva samples using cotton Salivette sampling devices (Sarstedt, Nümbrecht, Germany). Participants were asked to chew the swab from the Salivette for 60 s to activate salivation. The samples were stored frozen and later sent to the Dresden LabService GmbH for sAA analysis (Thoma et al., 2012).

### 2.5. Statistical Analyses

To investigate the tolerability and adverse effects of tVNS, separate *t*-tests comparing active stimulation and sham stimulation were conducted. To evaluate the effects of tVNS on heart rate, blood pressure, and sAA levels, separate analysis of variance (ANOVA) tests were carried out using the factors Time (pre, post) and Stimulation (active, sham). The log transformation was applied to the right-skewed sAA data to achieve a normal distribution. Repeated-measures ANOVA tests including the within-subject factor Valence (unpleasant, neutral, pleasant) and the between-subject factor Stimulation (active, sham) were used to investigate the effects of tVNS on the accuracy and response time of the lexical decision task. Non-words were not included in the analyses as they are of little theoretical importance. *Post hoc* analyses were conducted using the Bonferroni correction (Wright, 1992). Similarly, to assess the effects of tVNS on recognition memory, 3 (Valence) × 2 (Stimulation) repeated-measures ANOVA tests were used for the discrimination index *P*_*r*_ = *p*(hit) − *p*(false alarm) (Snodgrass and Corwin, 1988) and the bias index $B_{r}=\frac{p\left(\text{false alarm}\right)}{p\left(1-P_{r}\right)}$ (with *B*_*r*_ > 0.5 indicating liberal response bias and *B*_*r*_ < 0.5 conservative response bias). For the assessment of confidence in memory, analysis of the distribution of the confidence ratings revealed that a confidence rating of 5 occurred most frequently for correctly identified old words (hits) (40.73%). Evidence suggests that familiarity-based memory judgments increase gradually as a function of recognition confidence, whereas recollection-based memory judgments are generally associated with high-confidence memory judgments (Wixted and Stretch, 2004). Therefore, the proportion of hit rates based on their confidence ratings was calculated to assess the roles of recollection and familiarity in memory (Weymar et al., 2009; Rimmele et al., 2012). Those words that were correctly identified as old words and that received confidence ratings of 5 were classified into a *high-confidence hit rate* category, whereas all remaining words that were correctly identified as old words and that received lower confidence ratings were put into a *low-confidence hit rate* category. Recognition memory based on confidence ratings was analyzed using a three-way ANOVA with the within-subject factors Response Type (high-confidence hit rate, low-confidence hit rate) and Valence (unpleasant, neutral pleasant) and the between-subject factor Stimulation (active, sham).

## 3. Results

### 3.1. Side Effects of tVNS

Overall, subjective ratings indicated that the side effects of stimulation were low (*N* = 60, *M* = 2.02, SD = 0.88); see Table 1. Statistical analyses of subjective ratings indicated no significant differences between the active stimulation condition and the sham stimulation condition in any of the symptoms assessed (*ps* > 0.09), suggesting that side effects were minimal and comparable for the two types of stimulation.

**Table 1:** Mean subjective ratings (with standard errors in parentheses) for the stimulation side effects in the active stimulation condition and the sham stimulation condition.

	**Active**	**Sham**	**p-value**
Headache	1.83 (1.42)	2.03 (1.38)	0.58
Nausea	1.17 (0.75)	1.30 (0.75)	0.49
Dizziness	1.67 (1.15)	2.27 (1.55)	0.09
Neck pain	1.80 (1.49)	1.30 (0.75)	0.11
Neck contraction	2.27 (1.87)	1.93 (1.46)	0.44
Stinging sensation	2.87 (1.94)	2.80 (1.73)	0.93
Ear irritation	1.77 (1.45)	1.93 (1.55)	0.67
Concentration	2.97 (1.65)	3.47 (1.68)	0.25
Fluctuation of feelings	1.53 (1.01)	1.73 (1.34)	0.51
Unpleasant feelings	1.67 (1.15)	2.23 (1.45)	0.10

*Ratings were scored on a seven-point scale, with 1 being not at all and 7 very much*.

### 3.2. Autonomic Results

Table 2 provides an overview of the outcomes for the autonomic measures and salivary data during encoding. Statistical analyses revealed a main effect of Time for systolic blood pressure [*F*_(1, 114)_ = 6.23, *p* = 0.01, $\eta_{\text{p}}^{2}=0.05$], which suggests a decrease during encoding. This effect was not observed for diastolic blood pressure [*F*_(1, 114)_ = 2.23, *p* = 0.14, $\eta_{\text{p}}^{2}=0.02$] or for heart rate (*F* < 1). No main effect of Stimulation was observed for either systolic blood pressure (*F* < 1), diastolic blood pressure (*F* < 1), or heart rate (*F* < 1). No interaction effect of Time and Stimulation was found for either systolic blood pressure (*F* < 1), diastolic blood pressure (*F* < 1), or heart rate (*F* < 1), suggesting that stimulation had no significant impact on the autonomic measures over time. For sAA levels, no significant main effect of Time [*F*_(1, 58)_ = 2.1, *p* = 0.14, $\eta_{\text{p}}^{2}=0.03$], Stimulation (*F* < 1), or interaction between Time and Stimulation (*F* < 1) was found.

**Table 2:** Means (with standard deviations in parentheses) of the autonomic and salivary measures before and after active stimulation and sham stimulation in the encoding session (lexical decision task).

		**Heart rate**	**Systolic blood pressure**	**Diastolic blood pressure**	**Alpha-amylase**
		**(bpm)**	**(mmHg)**	**(mmHg)**	**[log(μkatal/l)]**
Active					
	Pre	73.97 (13.98)	116.13 (11.48)	77.43 (8.33)	4.46 (0.71)
	Post	71.10 (8.99)	107.97 (10.66)	73.40 (7.62)	4.46 (0.64)
Sham					
	Pre	72.73 (11.97)	116.10 (9.76)	77.93 (7.14)	4.52 (0.84)
	Post	70.33 (7.93)	108.50 (12.07)	73.80 (6.48)	4.24 (0.91)

### 3.3. Behavioral Results

#### 3.3.1. Lexical Decision Task

**Figure 2:**
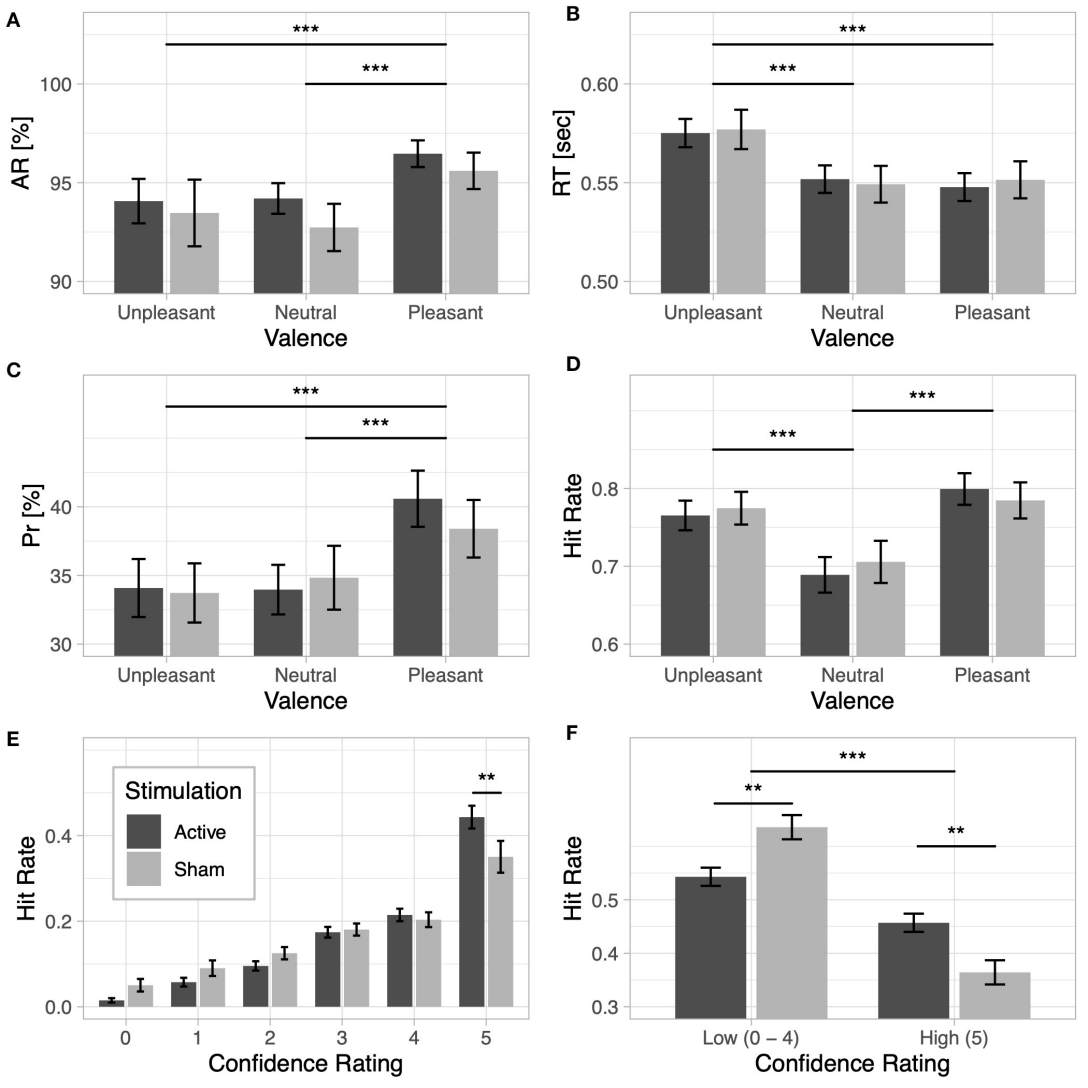
Lexical decision task: **(A)** accuracy rate (AR) and **(B)** response time (RT) for unpleasant, neutral, and pleasant words under the active and sham stimulation conditions. Recognition memory: **(C)** discrimination index (Pr) and **(D)** hit rate based on valence for the active and sham stimulation conditions. **(E)** Recognition confidence: hit rate based on confidence responses for the active and sham stimulation conditions. **(F)** Recognition memory based on confidence ratings: hit rate based on high-confidence category (words that received confidence ratings of 5) and low-confidence category (words that received confidence ratings from 0 to 4) for the active and sham stimulation conditions; note that the low-confidence hit rate category is the cumulative hit rate for ratings 0–4. Two asterisks indicate a *p*-value smaller than 0.01 and three asterisks indicate a *p*-value smaller than 0.001.

Behavioral results from the lexical decision task are presented in Table 3. Detection accuracy was modulated by the emotional content of words [*F*_(2, 116)_ = 12.62, *p* < 0.001, $\eta_{\text{p}}^{2}=0.18$] and was higher for pleasant words than for unpleasant words [*t*(59) = −3.38, *p* < 0.001, *d* = −0.29] and neutral words [*t*(59) = 5.13, *p* < 0.001, *d* = 0.5]. No differences were observed between unpleasant and neutral words [*t*(59) = 0.63, *p* = 0.53, *d* = 0.03]; see Figure 2A. No main effect of Stimulation (*F* < 1) or interaction with Valence (*F* < 1) was observed. For response times, a significant main effect of Valence [$F_{\left(2,\;116\right)}=48.06,p<0.001,\eta_{\text{p}}^{2}=0.45$] revealed longer response times for unpleasant words than for pleasant words [*t*(59) = 8.04, *p* < 0.001, *d* = 0.57] and neutral words [*t*(59) = 8.34, *p* < 0.001, *d* = 0.55]; no difference in response times was found between pleasant and neutral words [*t*(59) = −0.32, *p* = 0.75, *d* = −0.02; see Figure 2B]. No main effect of Stimulation (*F* < 1) or interaction (F < 1) was observed.

**Table 3:** Means (with standard deviations in parentheses) of the performance in the lexical decision task as a function of valence and stimulation.

		**Lexical decision task**		
		**Hits**	**False alarms**	**Response time (s)**
Active				
	Unpleasant	0.94 (0.06)	0.03 (0.02)	0.57 (0.10)
	Neutral	0.94 (0.04)	0.02 (0.02)	0.55 (0.09)
	Pleasant	0.96 (0.04)	0.03 (0.02)	0.55 (0.09)
Sham				
	Unpleasant	0.93 (0.09)	0.05 (0.04)	0.57 (0.11)
	Neutral	0.93 (0.06)	0.03 (0.04)	0.55 (0.09)
	Pleasant	0.96 (0.05)	0.04 (0.04)	0.55 (0.09)

#### 3.3.2. Recognition Memory

Results from the behavioral performance in the explicit recognition task are presented in Table 4. Memory accuracy, as measured by the discrimination index *P*_*r*_, was modulated by the emotional content of words [$F_{\left(2,\;116\right)}=10.65,p<0.001,\eta_{\text{p}}^{2}=0.15$]. Pleasant words were better discriminated than unpleasant [*t*(59) = −3.95, *p* < 0.001, *d* = −0.5] and neutral words [*t*(59) = 4.39, *p* < 0.001, *d* = 0.45]; no difference in *P*_*r*_ was observed between unpleasant and neutral words [*t*(59) = −0.35, *p* = 0.73, *d* = −0.04; see Figure 2C]. No main effect of Stimulation (*F* < 1) or interaction (F < 1) was found, however. For the bias index *B*_*r*_, a main effect of Valence was observed [$F_{\left(2,\;116\right)}=40.91,p<0.001,\eta_{\text{p}}^{2}=0.41$], indicating a more liberal response bias for emotional than for neutral words (*ps* < 0.001). No main effect of Stimulation or interaction (F < 1) was observed. A main effect of Valence was also observed for hit rates [$F_{\left(2,\;116\right)}=44.83,p<0.001,\eta_{\text{p}}^{2}=0.44$], revealing increased hit rates for both unpleasant [*t*(59) = 6.41, *p* < 0.001, *d* = 0.56] and pleasant words [*t*(59) = 9.25, *p* < 0.001, *d* = 0.72] compared to neutral words (see Figure 2D).

**Table 4:** Means (with standard deviations in parentheses) of the recognition memory performance and hit rate confidence ratings as a function of valence and stimulation.

		**Recognition memory**				**Recognition memory based on confidence ratings**	
		**Hits**	**False alarms**	***P*_*r*_** **index**	***B*_*r*_** **index**	**Familiarity-based hit rate**	**Recollection-based hit rate**
Active							
	Unpleasant	0.76 (0.10)	0.42 (0.11)	0.34 (0.12)	0.64 (0.15)	0.51 (0.16)	0.49 (0.16)
	Neutral	0.69 (0.12)	0.35 (0.10)	0.34 (0.10)	0.54 (0.15)	0.61 (0.15)	0.38 (0.15)
	Pleasant	0.80 (0.11)	0.39 (0.11)	0.41 (0.11)	0.66 (0.17)	0.50 (0.15)	0.50 (0.15)
Sham							
	Unpleasant	0.77 (0.11)	0.44 (0.13)	0.34 (0.12)	0.66 (0.16)	0.62 (0.22)	0.38 (0.22)
	Neutral	0.71 (0.15)	0.36 (0.10)	0.35 (0.13)	0.56 (0.17)	0.70 (0.20)	0.30 (0.20)
	Pleasant	0.78 (0.13)	0.40 (0.12)	0.38 (0.11)	0.66 (0.18)	0.59 (0.21)	0.41 (0.21)

#### 3.3.3. Recognition Memory Based on Confidence Ratings

When the proportion of hit rates based on subjects' confidence ratings were taken into account, a main effect of Response Type [$F_{\left(1,\;58\right)}=15.02,p<0.001,\eta_{\text{p}}^{2}=0.76$] and a significant interaction between Response Type and Stimulation [$F_{\left(1,\;58\right)}=4.04,p=0.049,\eta_{\text{p}}^{2}=0.46$] were observed, showing that active stimulation increased hit rates for words that were remembered with higher subjective confidence, as opposed to sham stimulation [*t*(58) = 2.03, *p* = 0.046, *d* = 0.52]. The reverse effect was observed for trials with low recognition confidence [*t*(58) = −2.03, *p* = 0.046, *d* = −0.52]; see Figures 2E,F. The results revealed a significant interaction of Response Type and Valence [$F_{\left(1,\;116\right)}=54.09,p<0.001,\eta_{\text{p}}^{2}=0.48$], indicating increased high-confidence hit rates for both unpleasant words [*t*(59) = −7.36, *p* < 0.001, *d* = −0.48] and pleasant words [*t*(59) = −11.03, *p* < 0.001, *d* = −0.59] compared to neutral words. The reverse effect was observed for trials with low recognition confidence, which showed increased hit rates for neutral words remembered with low confidence than for unpleasant [*t*(59) = 7.36, *p* < 0.001, *d* = 0.48] or pleasant words [*t*(59) = 11.03, *p* < 0.001, *d* = 0.59].

## 4. Discussion

In the present study, we investigated the impact of tVNS on emotional word processing (lexical decision task) and later recognition memory (old/new task). As an indirect marker of noradrenergic activation, sAA levels were measured before and after tVNS. As expected, emotion modulated word processing and memory. Pleasant words were better identified and remembered than neutral and unpleasant ones. However, tVNS showed no effects on word processing and overall emotional recognition memory performance. In addition, tVNS did not produce the expected sAA level increase, compared to sham stimulation. However, when high and low confidence ratings were considered, tVNS, compared to sham stimulation, increased the proportion of hit rates for words that were remembered with high confidence, irrespective of emotional category, suggesting an effect of tVNS on recollection-based memory performance.

### 4.1. Effects of Emotion on Word Processing and Recognition Memory but No Effect of tVNS

Replicating prior studies using lexical decision tasks (Kanske and Kotz, 2007; Schacht and Sommer, 2009; Scott et al., 2009; Kousta et al., 2011), we found that emotional word contents were better accessed than neutral ones. In the current study, however, we found an advantage for pleasant words, reflected in higher detection accuracy and shorter response times than for unpleasant words. Valence differences in word processing have also been reported in previous studies employing similar tasks (Herbert et al., 2006; Estes and Adelman, 2008; Nasrallah et al., 2009; Kissler and Koessler, 2011; Citron et al., 2014). It has been proposed that word frequency (Kahan and Hely, 2008; Scott et al., 2009) is one of the most important factors influencing emotional effects on word recognition, which may explain the amount of variance in word recognition latencies and accuracies across studies (for a review see Kuperman et al., 2014). Some authors argue that although emotional (both pleasant and unpleasant) words convey emotionally salient information, particularly unpleasant stimuli capture and hold attention in early processing because of their potentially threatening nature and relevance to survival (see the automatic vigilance hypothesis of Pratto and John, 1991). Therefore it could be that, in comparison with pleasant and neutral material, unpleasant words engage more attentional resources, thereby reducing the amount of resources available for lexical decision processes. Alternatively, however, because detection accuracy and response times were observed to be faster for pleasant than for unpleasant material (in line with Herbert et al., 2006; Estes and Adelman, 2008; Kissler et al., 2009; Kissler and Koessler, 2011; Citron et al., 2014), this could also indicate facilitated processing for positive compared to neutral concepts, either owing to higher interconnectivity in the mental lexicon (i.e., mental dictionary that contains information such as word meaning and syntactic characteristics, among other aspects) (Ashby et al., 1999) or, which may be more likely, because pleasant words are perceived as being more motivationally relevant than unpleasant ones (Citron et al., 2014). Particularly during low levels of emotional activation or arousal (see the theory of Cacioppo et al., 1999), there seems to be a preference for positive information that mediates approach motivation (in contrast to a preference for negative information that leads to defense motivation at high levels of arousal). This latter theoretical view seems reasonable for explaining the present results, since induced arousal is often lower for words than for faces or scenes (see also the recent ERP evidence in Bayer and Schacht, 2014, showing early positivity bias for words as compared to faces and scenes).

We also found better long-term memory discrimination for pleasant words than for the other semantic categories. Given the results from the lexical decision task, this indicates that prioritized processing during encoding for pleasant contents may have promoted deeper consolidation processes, which produced enhanced long-term memory performance (Weymar et al., 2012; for a review see Cross et al., 2018). As for the encoding data, previous research on recognition memory also found a memory advantage for pleasant words (Herbert et al., 2008). However, it should be noted that arousal-specific effects or even unpleasant enhancing effects have also been reported (Kensinger and Corkin, 2003; Weymar and Hamm, 2013; Weymar et al., 2014). The inconsistent effects of arousal and valence on recognition memory across studies may, however, be a result of differences in arousal and valence levels of the stimuli used in experiments and may also be related to differences in lexico-semantic variables, such as the frequency, concreteness, imageability, age of acquisition, and familiarity of words (Scott et al., 2009; Kousta et al., 2011), which also likely lead to differences in word processing studies. Interestingly, though, besides valence-specific effects in overall memory, we found enhanced memory for both pleasant and unpleasant words when high-confidence responses were taken into account, which replicates many studies showing that the memory-enhancing effect of emotion is mediated by the process of recollection rather than familiarity (Ochsner, 2000; Kensinger and Corkin, 2003; Dolcos et al., 2004, 2005; Sharot et al., 2004, 2007; Weymar et al., 2009; Rimmele et al., 2012). The results of the present study also show that qualitative memory retrieval does not necessarily depend on the level of word processing during encoding (lexical decision task).

Critically, and relevant to the main research question, active tVNS, as compared to sham stimulation, did not affect lexical decision performance and recognition memory performance for emotional and neutral words, which is contrary to our expectations. From animal models it has been suggested that stimulation of vagal afferents leads to activation of the LC-NE system, which increases arousal levels in the brain. Arousal, in turn, was expected to directly facilitate amygdala function, by promoting on the one hand the processing of highly relevant information but also by influencing regions that support memory consolidation, such as the hippocampus (Mather and Sutherland, 2011; Mather et al., 2016). In contrast to prior studies (Ventura-Bort et al., 2018; Warren et al., 2019), however, we found no evidence in the overall analysis of enhanced sAA levels after tVNS, compared to sham stimulation (Koenig et al., 2019). Assuming that changes in sAA levels reflect changes in central noradrenergic levels (Chatterton et al., 1996; Warren et al., 2017), the lack of an increase in the sAA level may point toward an insufficient activation of the arousal-modulated LC-NE system in the present study that made enhanced emotional word processing and subsequent memory storage unlikely. Similarly, although our material was emotionally laden, as indicated by the ratings and the expected enhancement observed in the lexical decision and recognition memory tasks, arousal levels are usually lower for words and faces than for affective scenes (Lang et al., 1998; Bradley, 2000). Thus, emotional words themselves may not have sufficiently triggered arousal to produce a significant impact of tVNS on processing and memory. This argument is also substantiated by preliminary data from our lab (Weymar et al., 2019) showing that tVNS can indeed facilitate recognition memory when emotional scenes are used, which was also found to be related to an increase in sAA levels. Another potentially important factor that may have led to no changes in sAA levels relates to the stimulation duration and protocol of the current study. Prior studies showing an increase in sAA levels after tVNS used continuous stimulation or stimulated for longer periods of time (more than the 23 min in the current study) (Ventura-Bort et al., 2018; Warren et al., 2019). It could therefore be that the stimulation protocol used in the current study was not optimal for increasing arousal as reflected in changes of sAA levels. It should be noted, however, that, irrespective of a potential central noradrenergic activation by tVNS, the reliability of elevated alpha-amylase levels as a biomarker (for recent findings see Warren et al., 2017) has not yet been sufficiently proven (e.g., Nater and Rohleder, 2009; Bosch et al., 2011). Therefore, additional variables are clearly needed to study the involvement of NE in emotion-cognition interactions.

### 4.2. Effects of tVNS on Recollection-Based Memory

Despite finding no tVNS effects on overall memory, tVNS modulated recognition confidence. When the proportion of hit rates based on low and high confidence ratings were considered, reflecting familiarity- and recollection-based memory (Wixted and Stretch, 2004), respectively, higher recollection performance was observed during tVNS compared to sham stimulation, partly supporting our a priori hypothesis. This result may indicate that tVNS facilitates memory consolidation, resulting in a greater recollective experience of past memories irrespective of their emotional category. This finding is partially in line with the clinical study of Clark et al. (1999) using invasive VNS in epileptic patients, which showed better memory for words, suggesting that vagus nerve activation modulates word memory formation in humans.

Previous studies have shown that the hippocampus plays an important role in recollection (for a review see Brown and Aggleton, 2001, or Eichenbaum et al., 2007). For instance, patients with selective hippocampal damage exhibit more pronounced deficits on tasks related to recollection processes, such as associative and source memory tasks (Holdstock et al., 2005; Gold et al., 2006; for a review see Yonelinas et al., 2010). Further evidence comes from animal studies showing that selective hippocampal damage impaired recollection but spared familiarity-based odor recognition in rats (Fortin et al., 2004; Sauvage et al., 2008; for a review see Rugg and Vilberg, 2013) and from fMRI studies showing hippocampal involvement primarily in recollection processes in healthy humans (Brown and Aggleton, 2001; Ranganath and Ritchey, 2012; for a review see Eichenbaum et al., 2007). The enhanced recollection-based memory for items encoded under tVNS may therefore indicate that tVNS modulates, to some extent, hippocampal activation (Roosevelt et al., 2006; Raedt et al., 2011). Although the underlying neural circuitry is not well-understood, previous research has shown that tVNS most likely activates NE secretion in the LC, which projects to a wide variety of cortical and subcortical regions, including not only the amygdala but also the hippocampus (Van Bockstaele et al., 1999; McIntyre et al., 2012). Giving support to this assumption, prior research in animals using implanted vagus nerve stimulators found that invasive VNS increases LC firing rates in rats (Dorr and Debonnel, 2006), hippocampal activity (Roosevelt et al., 2006; Raedt et al., 2011), and long-term memory for inhibitory avoidance (Clark et al., 1998). Similarly, Jacobs et al. (2015) found that tVNS, compared to sham simulation, increases associative memory in older humans. Although the noradrenergic activation of the amygdala is fundamental for memory-enhancing effects of emotion, recent studies indicate that the LC can facilitate memory processes through an amygdala-independent path, projecting directly to the hippocampus (Mello-Carpes and Izquierdo, 2013). It might therefore be that the non-specific effects of tVNS on word recollection memory are due to a lesser involvement of the amygdala in the hippocampus-mediated memory consolidation process (which may partly be driven by the nature of the stimulus material; see Kensinger and Schachter, 2006). Altogether, the current results indicate that tVNS can facilitate hippocampus-mediated, recollection-based memory.

### 4.3. Conclusion

To summarize, the present study has replicated prior research showing enhanced processing and memory for emotional (pleasant) words (particularly when based on high subjective confidence). Although tVNS showed no effects on word processing, subtle effects on recollection-based memory (high-confidence memory) were found, which may indicate that tVNS facilitates hippocampus-mediated consolidation processes. The potential of tVNS to improve memory in individuals (cf. Jacobs et al., 2015) supports its relevance for future research. For example, tVNS may be used to enhance memory consolidation during sleep, which is also related to hippocampus (Moroni et al., 2007) and LC activity (Eschenko et al., 2012). Furthermore, tVNS may be relevant in clinical applications, for instance as a therapeutic tool (and in combination with emerging neuroscientific approaches; see e.g., Dennis and Thompson, 2014 and Vecchio et al., 2015) in cognitive aging and for the treatment of a number of neuropsychiatric disorders associated with cognitive impairment, such as depression or Alzheimer's disease (Broncel et al., 2020).

## Data Availability Statement

The datasets generated for this study are available on request to the corresponding author.

## Ethics Statement

The studies involving human participants were reviewed and approved by Ethikkommission Universität Potsdam. The patients/participants provided their written informed consent to participate in this study.

## Author Contributions

MG and MW conceived the idea and discussed the design of the study. MG programmed the experiment and collected and analyzed the data. All authors discussed the results and contributed to writing the manuscript.

## Conflict of Interest

The authors declare that the research was conducted in the absence of any commercial or financial relationships that could be construed as a potential conflict of interest.

## References

[B1] Armony J. L. Dolan R. J. (2002). Modulation of spatial attention by fear-conditioned stimuli: an event-related fMRI study. Neuropsychologia 40, 817–826. 10.1016/S0028-3932(01)00178-6 11900732

[B2] Ashby F. G. Isen A. M. Turken A. U. (1999). A neuropsychological theory of positive affect and its influence on cognition. Psychol. Rev. 106:529. 10.1037/0033-295X.106.3.529 10467897

[B3] Bayer M. Schacht A. (2014). Event-related brain responses to emotional words, pictures, and faces-a cross-domain comparison. Front. Psychol. 5:1106. 10.3389/fpsyg.2014.01106 25339927 PMC4186271

[B4] Bosch J. A. Veerman E. C. de Geus E. J. Proctor G. B. (2011). α-amylase as a reliable and convenient measure of sympathetic activity: don't start salivating just yet! Psychoneuroendocrinology 36, 449–453. 10.1016/j.psyneuen.2010.12.019 21295411

[B5] Bradley M. M. (2000). Emotion and motivation, in Handbook of Psychophysiology, eds Cacioppo J. T. Tassinary L. G. Berntson G. G. (Cambridge University Press), 602–642.

[B6] Bradley M. M. Greenwald M. K. Petry M. C. Lang P. J. (1992). Remembering pictures: pleasure and arousal in memory. J. Exp. Psychol. Learn. Mem. Cogn. 18:379. 10.1037/0278-7393.18.2.379 1532823

[B7] Broncel A. Bocian R. Kłos-Wojtczak P. Kulbat-Warycha K. Konopacki J. (2020). Vagal nerve stimulation as a promising tool in the improvement of cognitive disorders. Brain Res. Bull. 155, 37–47. 10.1016/j.brainresbull.2019.11.011 31790720

[B8] Brown M. W. Aggleton J. P. (2001). Recognition memory: what are the roles of the perirhinal cortex and hippocampus? Nat. Rev. Neurosci. 2:51. 10.1038/35049064 11253359

[B9] Burger A. M. Verkuil B. Van Diest I. Van der Does W. Thayer J. F. Brosschot J. F. (2016). The effects of transcutaneous vagus nerve stimulation on conditioned fear extinction in humans. Neurobiol. Learn. Mem. 132, 49–56. 10.1016/j.nlm.2016.05.007 27222436

[B10] Cacioppo J. T. Gardner W. L. Berntson G. G. (1999). The affect system has parallel and integrative processing components: form follows function. J. Pers. Soc. Psychol. 76:839 10.1037/0022-3514.76.5.839

[B11] Cahill L. McGaugh J. L. (1995). A novel demonstration of enhanced memory associated with emotional arousal. Conscious. Cogn. 4, 410–421. 10.1006/ccog.1995.1048 8750416

[B12] Cahill L. McGaugh J. L. (1998). Mechanisms of emotional arousal and lasting declarative memory. Trends Neurosci. 21, 294–299. 10.1016/S0166-2236(97)01214-9 9683321

[B13] Chatterton R. T. Jr. Vogelsong K. M. Lu Y.-C. Ellman A. B. Hudgens G. A. (1996). Salivary α-amylase as a measure of endogenous adrenergic activity. Clin. Physiol. 16, 433–448. 10.1111/j.1475-097X.1996.tb00731.x 8842578

[B14] Citron F. M. Weekes B. S. Ferstl E. C. (2014). Arousal and emotional valence interact in written word recognition. Lang. Cogn. Neurosci. 29, 1257–1267. 10.1080/23273798.2014.897734

[B15] Clark K. Smith D. Hassert D. Browning R. Naritoku D. Jensen R. (1998). Posttraining electrical stimulation of vagal afferents with concomitant vagal efferent inactivation enhances memory storage processes in the rat. Neurobiol. Learn. Mem. 70, 364–373. 10.1006/nlme.1998.3863 9774527

[B16] Clark K. B. Naritoku D. K. Smith D. C. Browning R. A. Jensen R. A. (1999). Enhanced recognition memory following vagus nerve stimulation in human subjects. Nat. Neurosci. 2:94. 10.1038/4600 10195186

[B17] Clewett D. V. Huang R. Velasco R. Lee T.-H. Mather M. (2018). Locus coeruleus activity strengthens prioritized memories under arousal. J. Neurosci. 38, 1558–1574. 10.1523/JNEUROSCI.2097-17.2017 29301874 PMC5815354

[B18] Colzato L. S. Sellaro R. Beste C. (2017). Darwin revisited: The vagus nerve is a causal element in controlling recognition of other's emotions. Cortex 92, 95–102. 10.1016/j.cortex.2017.03.017 28460255

[B19] Cross Z. R. Santamaria A. Kohler M. (2018). Attention and emotion-enhanced memory: a systematic review and meta-analysis of behavioural and neuroimaging evidence. bioRxiv [Preprint] 273920. 10.1101/273920

[B20] D'Argembeau A. Van der Linden M. (2004). Influence of affective meaning on memory for contextual information. Emotion 4:173. 10.1037/1528-3542.4.2.173 15222854

[B21] Dennis E. L. Thompson P. M. (2014). Functional brain connectivity using fMRI in aging and alzheimer's disease. Neuropsychol. Rev. 24, 49–62. 10.1007/s11065-014-9249-6 24562737 PMC4109887

[B22] Dietrich S. Smith J. Scherzinger C. Hofmann-Preiß K. Freitag T. Eisenkolb A. (2008). A novel transcutaneous vagus nerve stimulation leads to brainstem and cerebral activations measured by functional MRI/funktionelle magnetresonanztomographie zeigt aktivierungen des hirnstamms und weiterer zerebraler strukturen unter transkutaner vagusnervstimulation. Biomed. Eng. 53, 104–111. 10.1515/BMT.2008.022 18601618

[B23] Doerksen S. Shimamura A. P. (2001). Source memory enhancement for emotional words. Emotion 1:5. 10.1037/1528-3542.1.1.5 12894807

[B24] Dolan R. J. (2002). Emotion, cognition, and behavior. Science 298, 1191–1194. 10.1126/science.1076358 12424363

[B25] Dolcos F. Cabeza R. (2002). Event-related potentials of emotional memory: encoding pleasant, unpleasant, and neutral pictures. Cogn. Affect. Behav. Neurosci. 2, 252–263. 10.3758/CABN.2.3.252 12775189

[B26] Dolcos F. Katsumi Y. Bogdan P. Shen C. Jun S. Buetti S.. (2020a). The impact of focused attention on subsequent emotional recollection: a functional MRI investigation. Neuropsychologia 138:107338. 10.1016/j.neuropsychologia.2020.107338 31926178

[B27] Dolcos F. Katsumi Y. Moore M. Berggren N. de Gelder B. Derakshan N.. (2020b). Neural correlates of emotion-attention interactions: from perception, learning, and memory to social cognition, individual differences, and training interventions. Neurosci. Biobehav. Rev. 108, 559–601. 10.1016/j.neubiorev.2019.08.017 31446010

[B28] Dolcos F. Katsumi Y. Weymar M. Moore M. Tsukiura T. Dolcos S. (2017). Emerging directions in emotional episodic memory. Front. Psychol. 8:1867. 10.3389/fpsyg.2017.01867 29255432 PMC5723010

[B29] Dolcos F. LaBar K. S. Cabeza R. (2004). Interaction between the amygdala and the medial temporal lobe memory system predicts better memory for emotional events. Neuron 42, 855–863. 10.1016/S0896-6273(04)00289-2 15182723

[B30] Dolcos F. LaBar K. S. Cabeza R. (2005). Remembering one year later: role of the amygdala and the medial temporal lobe memory system in retrieving emotional memories. Proc. Natl. Acad. Sci. U.S.A. 102, 2626–2631. 10.1073/pnas.0409848102 15703295 PMC548968

[B31] Dorr A. E. Debonnel G. (2006). Effect of vagus nerve stimulation on serotonergic and noradrenergic transmission. J. Pharmacol. Exp. Therapeut. 318, 890–898. 10.1124/jpet.106.104166 16690723

[B32] Eichenbaum H. Yonelinas A. P. Ranganath C. (2007). The medial temporal lobe and recognition memory. Annu. Rev. Neurosci. 30, 123–152. 10.1146/annurev.neuro.30.051606.094328 17417939 PMC2064941

[B33] Ellrich J. (2011). Transcutaneous vagus nerve stimulation. Eur Neurol Rev 6, 262–264. 10.17925/ENR.2011.06.04.254

[B34] Eschenko O. Magri C. Panzeri S. Sara S. J. (2012). Noradrenergic neurons of the locus coeruleus are phase locked to cortical up-down states during sleep. Cereb. Cortex 22, 426–435. 10.1093/cercor/bhr121 21670101

[B35] Estes Z. Adelman J. S. (2008). Automatic vigilance for negative words in lexical decision and naming: comment on larsen, mercer, and balota (2006). Emotion 8, 441–444. 10.1037/1528-3542.8.4.441 18729575

[B36] Eviatar Z. Zaidel E. (1991). The effects of word length and emotionality on hemispheric contribution to lexical decision. Neuropsychologia 29, 415–428. 10.1016/0028-3932(91)90028-7 1886683

[B37] Fischer R. Ventura-Bort C. Hamm A. Weymar M. (2018). Transcutaneous vagus nerve stimulation (TVNS) enhances conflict-triggered adjustment of cognitive control. Cogn. Affect. Behav. Neurosci. 18, 680–693. 10.3758/s13415-018-0596-2 29693214

[B38] Fortin N. J. Wright S. P. Eichenbaum H. (2004). Recollection-like memory retrieval in rats is dependent on the hippocampus. Nature 431, 188–191. 10.1038/nature02853 15356631 PMC4053162

[B39] Fox E. Lester V. Russo R. Bowles R. Pichler A. Dutton K. (2000). Facial expressions of emotion: Are angry faces detected more efficiently? Cogn. Emot. 14, 61–92. 10.1080/026999300378996 17401453 PMC1839771

[B40] Gold J. J. Hopkins R. O. Squire L. R. (2006). Single-item memory, associative memory, and the human hippocampus. Learn. Mem. 13, 644–649. 10.1101/lm.258406 16980546 PMC1635407

[B41] Groch S. Wilhelm I. Diekelmann S. Sayk F. Gais S. Born J. (2011). Contribution of norepinephrine to emotional memory consolidation during sleep. Psychoneuroendocrinology 36, 1342–1350. 10.1016/j.psyneuen.2011.03.006 21493010

[B42] Hamann S. B. Ely T. D. Grafton S. T. Kilts C. D. (1999). Amygdala activity related to enhanced memory for pleasant and aversive stimuli. Nat. Neurosci. 2:289. 10.1038/6404 10195224

[B43] Hassert D. Miyashita T. Williams C. (2004). The effects of peripheral vagal nerve stimulation at a memory-modulating intensity on norepinephrine output in the basolateral amygdala. Behav. Neurosci. 118:79. 10.1037/0735-7044.118.1.79 14979784

[B44] Herbert C. Junghofer M. Kissler J. (2008). Event related potentials to emotional adjectives during reading. Psychophysiology 45, 487–498. 10.1111/j.1469-8986.2007.00638.x 18221445

[B45] Herbert C. Kissler J. Junghöfer M. Peyk P. Rockstroh B. (2006). Processing of emotional adjectives: evidence from startle EMG and ERPs. Psychophysiology 43, 197–206. 10.1111/j.1469-8986.2006.00385.x 16712590

[B46] Holdstock J. Mayes A. Gong Q. Roberts N. Kapur N. (2005). Item recognition is less impaired than recall and associative recognition in a patient with selective hippocampal damage. Hippocampus 15, 203–215. 10.1002/hipo.20046 15390152

[B47] Jacobs H. I. Riphagen J. M. Razat C. M. Wiese S. Sack A. T. (2015). Transcutaneous vagus nerve stimulation boosts associative memory in older individuals. Neurobiol. Aging 36, 1860–1867. 10.1016/j.neurobiolaging.2015.02.023 25805212

[B48] Kahan T. A. Hely C. D. (2008). The role of valence and frequency in the emotional stroop task. Psychon. Bull. Rev. 15, 956–960. 10.3758/PBR.15.5.956 18926988

[B49] Kanske P. Kotz S. A. (2007). Concreteness in emotional words: ERP evidence from a hemifield study. Brain Res. 1148, 138–148. 10.1016/j.brainres.2007.02.044 17391654

[B50] Kensinger E. A. (2009). What factors need to be considered to understand emotional memories? Emot. Rev. 1, 120–121. 10.1177/1754073908100436 19655033 PMC2719895

[B51] Kensinger E. A. Corkin S. (2003). Memory enhancement for emotional words: are emotional words more vividly remembered than neutral words? Mem. Cogn. 31, 1169–1180. 10.3758/BF03195800 15058678

[B52] Kensinger E. A. Schachter D. L. (2006). Processing emotional pictures and words: effects of valence and arousal. Cogn. Affect. Behav. Neurosci. 6, 110–126. 10.3758/CABN.6.2.110 17007232

[B53] Keute M. Boehere L. Ruhnau P. Heinze H.-J. Zaehle T. (2019). Transcutaneous vagus nerve stimulation (TVNS) and the dynamics of visual bistable perception. Front. Neurosci. 13:227. 10.3389/fnins.2019.00227 30906250 PMC6418039

[B54] Kissler J. Herbert C. Winkler I. Junghofer M. (2009). Emotion and attention in visual word processing–an erp study. Biol. Psychol. 80, 75–83. 10.1016/j.biopsycho.2008.03.004 18439739

[B55] Kissler J. Koessler S. (2011). Emotionally positive stimuli facilitate lexical decisions–an ERP study. Biol. Psychol. 86, 254–264. 10.1016/j.biopsycho.2010.12.006 21184799

[B56] Koenig J. Parzer P. Haigis N. Liebemann J. Jung T. Resch F.. (2019). Effects of acute transcutaneous vagus nerve stimulation on emotion recognition in adolescent depression. Psychol. Med. 10.1017/S0033291719003490. [Epub ahead of print]. PMC7958483 31818339

[B57] Kousta S.-T. Vigliocco G. Vinson D. P. Andrews M. Del Campo E. (2011). The representation of abstract words: why emotion matters. J. Exp. Psychol. Gen. 140:14. 10.1037/a0021446 21171803

[B58] Kraus T. Kiess O. Hösl K. Terekhin P. Kornhuber J. Forster C. (2013). CNS bold fMRI effects of sham-controlled transcutaneous electrical nerve stimulation in the left outer auditory canal-a pilot study. Brain Stimul. 6, 798–804. 10.1016/j.brs.2013.01.011 23453934

[B59] Kuperman V. Estes Z. Brysbaert M. Warriner A. B. (2014). Emotion and language: valence and arousal affect word recognition. J. Exp. Psychol. Gen. 143:1065. 10.1037/a0035669 24490848 PMC4038659

[B60] Lang P. J. Bradley M. M. (2010). Emotion and the motivational brain. Biol. Psychol. 84, 437–450. 10.1016/j.biopsycho.2009.10.007 19879918 PMC3612949

[B61] Lang P. J. Bradley M. M. Cuthbert B. N. (1998). Emotion, motivation, and anxiety: brain mechanisms and psychophysiology. Biol. Psychiatry 44, 1248–1263. 10.1016/S0006-3223(98)00275-3 9861468

[B62] Lewine J. D. Paulson K. Bangera N. Simon B. J. (2019). Exploration of the impact of brief noninvasive vagal nerve stimulation on EEG and event-related potentials. Neuromodul. Technol. Neural Interface 22, 564–572. 10.1111/ner.12864 30288866

[B63] Mather M. Clewett D. Sakaki M. Harley C. W. (2016). Norepinephrine ignites local hot spots of neuronal excitation: How arousal amplifies selectivity in perception and memory. Behav. Brain Sci. 39:e200. 10.1017/S0140525X15000667 26126507 PMC5830137

[B64] Mather M. Sutherland M. R. (2011). Arousal-biased competition in perception and memory. Perspect. Psychol. Sci. 6, 114–133. 10.1177/1745691611400234 21660127 PMC3110019

[B65] McGaugh J. L. (2015). Consolidating memories. Annu. Rev. Psychol. 66, 1–24. 10.1146/annurev-psych-010814-014954 25559113

[B66] McIntyre C. K. McGaugh J. L. Williams C. L. (2012). Interacting brain systems modulate memory consolidation. Neurosci. Biobehav. Rev. 36, 1750–1762. 10.1016/j.neubiorev.2011.11.001 22085800 PMC3315607

[B67] Mello-Carpes P. B. Izquierdo I. (2013). The nucleus of the solitary tract → nucleus paragigantocellularis → locus coeruleus → ca1 region of dorsal hippocampus pathway is important for consolidation of object recognition memory. Neurobiol. Learn. Mem. 100, 56–63. 10.1016/j.nlm.2012.12.002 23246466

[B68] Mogg K. Bradley B. P. De Bono J. Painter M. (1997). Time course of attentional bias for threat information in non-clinical anxiety. Behav. Res. Therapy 35, 297–303. 10.1016/S0005-7967(96)00109-X 9134784

[B69] Moroni F. Nobili L. Curcio G. De Carli F. Fratello F. Marzano C.. (2007). Sleep in the human hippocampus: a stereo-EEG study. PLoS ONE 2:e867. 10.1371/journal.pone.0000867 17848998 PMC1959185

[B70] Nasrallah M. Carmel D. Lavie N. (2009). Murder, she wrote: enhanced sensitivity to negative word valence. Emotion 9:609. 10.1037/a0016305 19803583 PMC2759814

[B71] Nater U. M. Rohleder N. (2009). Salivary alpha-amylase as a non-invasive biomarker for the sympathetic nervous system: current state of research. Psychoneuroendocrinology 34, 486–496. 10.1016/j.psyneuen.2009.01.014 19249160

[B72] Nieuwenhuis S. Aston-Jones G. Cohen J. D. (2005). Decision making, the p3, and the locus coeruleus-norepinephrine system. Psychol. Bull. 131:510. 10.1037/0033-2909.131.4.510 16060800

[B73] Nishida M. Pearsall J. Buckner R. L. Walker M. P. (2009). REM sleep, prefrontal theta, and the consolidation of human emotional memory. Cereb. Cortex 19, 1158–1166. 10.1093/cercor/bhn155 18832332 PMC2665156

[B74] Ochsner K. N. (2000). Are affective events richly recollected or simply familiar? The experience and process of recognizing feelings past. J. Exp. Psychol. Gen. 129:242. 10.1037/0096-3445.129.2.242 10868336

[B75] Öhman A. Flykt A. Esteves F. (2001). Emotion drives attention: detecting the snake in the grass. J. Exp. Psychol. Gen. 130:466. 10.1037/0096-3445.130.3.466 11561921

[B76] Ortigue S. Michel C. M. Murray M. M. Mohr C. Carbonnel S. Landis T. (2004). Electrical neuroimaging reveals early generator modulation to emotional words. Neuroimage 21, 1242–1251. 10.1016/j.neuroimage.2003.11.007 15050552

[B77] Payne J. D. Stickgold R. Swanberg K. Kensinger E. A. (2008). Sleep preferentially enhances memory for emotional components of scenes. Psychol. Sci. 19, 781–788. 10.1111/j.1467-9280.2008.02157.x 18816285 PMC5846336

[B78] Peuker E. T. Filler T. J. (2002). The nerve supply of the human auricle. Clin. Anat. 15, 35–37. 10.1002/ca.1089 11835542

[B79] Phelps E. A. LaBar K. S. Spencer D. D. (1997). Memory for emotional words following unilateral temporal lobectomy. Brain Cogn. 35, 85–109. 10.1006/brcg.1997.0929 9339304

[B80] Pratto F. John O. P. (1991). Automatic vigilance: the attention-grabbing power of negative social information. J. Pers. Soc. Psychol. 61:380. 10.1037/0022-3514.61.3.380 1941510

[B81] Raedt R. Clinckers R. Mollet L. Vonck K. El Tahry R. Wyckhuys T.. (2011). Increased hippocampal noradrenaline is a biomarker for efficacy of vagus nerve stimulation in a limbic seizure model. J. Neurochem. 117, 461–469. 10.1111/j.1471-4159.2011.07214.x 21323924

[B82] Ranganath C. Ritchey M. (2012). Two cortical systems for memory-guided behaviour. Nat. Rev. Neurosci. 13:713. 10.1038/nrn3338 22992647

[B83] Righi S. Marzi T. Toscani M. Baldassi S. Ottonello S. Viggiano M. (2012). Fearful expressions enhance recognition memory: electrophysiological evidence. Acta Psychol. 139, 7–18. 10.1016/j.actpsy.2011.09.015 22036588

[B84] Rimmele U. Davachi L. Phelps E. A. (2012). Memory for time and place contributes to enhanced confidence in memories for emotional events. Emotion 12:834. 10.1037/a0028003 22642353 PMC3968901

[B85] Roosevelt R. W. Smith D. C. Clough R. W. Jensen R. A. Browning R. A. (2006). Increased extracellular concentrations of norepinephrine in cortex and hippocampus following vagus nerve stimulation in the rat. Brain Res. 1119, 124–132. 10.1016/j.brainres.2006.08.048 16962076 PMC1751174

[B86] Rufener K. S. Geyer U. Janitzky K. Heinze H.-J. Zaehle T. (2018). Modulating auditory selective attention by non-invasive brain stimulation: Differential effects of transcutaneous vagal nerve stimulation and transcranial random noise stimulation. Eur. J. Neurosci. 48, 2301–2309. 10.1111/ejn.14128 30144194

[B87] Rugg M. D. Vilberg K. L. (2013). Brain networks underlying episodic memory retrieval. Curr. Opin. Neurobiol. 23, 255–260. 10.1016/j.conb.2012.11.005 23206590 PMC3594562

[B88] Sauter D. A. Eimer M. (2010). Rapid detection of emotion from human vocalizations. J. Cogn. Neurosci. 22, 474–481. 10.1162/jocn.2009.21215 19302002

[B89] Sauvage M. M. Fortin N. J. Owens C. B. Yonelinas A. P. Eichenbaum H. (2008). Recognition memory: opposite effects of hippocampal damage on recollection and familiarity. Nat. Neurosci. 11, 16–18. 10.1038/nn2016 18037884 PMC4053160

[B90] Schacht A. Sommer W. (2009). Time course and task dependence of emotion effects in word processing. Cogn. Affect. Behav. Neurosci. 9, 28–43. 10.3758/CABN.9.1.28 19246325

[B91] Schupp H. T. Flaisch T. Stockburger J. Junghöfer M. (2006). Emotion and attention: event-related brain potential studies. Prog. Brain Res. 156, 31–51. 10.1016/S0079-6123(06)56002-9 17015073

[B92] Schupp H. T. Öhman A. Junghöfer M. Weike A. I. Stockburger J. Hamm A. O. (2004). The facilitated processing of threatening faces: an ERP analysis. Emotion 4:189. 10.1037/1528-3542.4.2.189 15222855

[B93] Scott G. G. O'Donnell P. J. Leuthold H. Sereno S. C. (2009). Early emotion word processing: evidence from event-related potentials. Biol. Psychol. 80, 95–104. 10.1016/j.biopsycho.2008.03.010 18440691

[B94] Sellaro R. de Gelder B. Finisguerra A. Colzato L. S. (2018). Transcutaneous vagus nerve stimulation (TVNS) enhances recognition of emotions in faces but not bodies. Cortex 99, 213–223. 10.1016/j.cortex.2017.11.007 29275193

[B95] Sellaro R. van Leusden J. W. Tona K.-D. Verkuil B. Nieuwenhuis S. Colzato L. S. (2015). Transcutaneous vagus nerve stimulation enhances post-error slowing. J. Cogn. Neurosci. 27, 2126–2132. 10.1162/jocn_a_00851 26226074

[B96] Sharot T. Delgado M. R. Phelps E. A. (2004). How emotion enhances the feeling of remembering. Nat. Neurosci. 7:1376. 10.1038/nn1353 15558065

[B97] Sharot T. Verfaellie M. Yonelinas A. P. (2007). How emotion strengthens the recollective experience: a time-dependent hippocampal process. PLoS ONE 2:e1068. 10.1371/journal.pone.0001068 17971848 PMC2031918

[B98] Snodgrass J. G. Corwin J. (1988). Pragmatics of measuring recognition memory: applications to dementia and amnesia. J. Exp. Psychol. Gen. 117:34. 10.1037/0096-3445.117.1.34 2966230

[B99] Steenbergen L. Sellaro R. Stock A.-K. Verkuil B. Beste C. Colzato L. S. (2015). Transcutaneous vagus nerve stimulation (TVNS) enhances response selection during action cascading processes. Eur. Neuropsychopharmacol. 25, 773–778. 10.1016/j.euroneuro.2015.03.015 25869158

[B100] Sterpenich V. D'Argembeau A. Desseilles M. Balteau E. Albouy G. Vandewalle G.. (2006). The locus ceruleus is involved in the successful retrieval of emotional memories in humans. J. Neurosci. 26, 7416–7423. 10.1523/JNEUROSCI.1001-06.2006 16837589 PMC6674193

[B101] Szeska C. Richter J. Wendt J. Weymar M. Hamm A. O. (2020). Promoting long-term inhibition of human fear responses by non-invasive transcutaneous vagus nerve stimulation during extinction training. Sci. Rep. 10, 1–16. 10.1038/s41598-020-58412-w 32001763 PMC6992620

[B102] Thoma M. V. Kirschbaum C. Wolf J. M. Rohleder N. (2012). Acute stress responses in salivary alpha-amylase predict increases of plasma norepinephrine. Biol. Psychol. 91, 342–348. 10.1016/j.biopsycho.2012.07.008 22954623

[B103] Van Bockstaele E. J. Peoples J. Telegan P. (1999). Efferent projections of the nucleus of the solitary tract to peri-locus coeruleus dendrites in rat brain: Evidence for a monosynaptic pathway. J. Comp. Neurol. 412, 410–428. 10.1002/(SICI)1096-9861(19990927)412:3<410::AID-CNE3>3.0.CO;2-F 10441230

[B104] Van Leusden J. W. Sellaro R. Colzato L. S. (2015). Transcutaneous vagal nerve stimulation (TVNS): a new neuromodulation tool in healthy humans? Front. Psychol. 6:102. 10.3389/fpsyg.2015.00102 25713547 PMC4322601

[B105] Vecchio F. Miraglia F. Curcio G. Altavilla R. Scrascia F. Giambattistelli F.. (2015). Cortical brain connectivity evaluated by graph theory in dementia: a correlation study between functional and structural data. J. Alzheimers Dis. 45, 745–756. 10.3233/JAD-142484 25613102

[B106] Ventura-Bort C. Wirkner J. Genheimer H. Wendt J. Hamm A. O. Weymar M. (2018). Effects of transcutaneous vagus nerve stimulation (TVNS) on the p300 and alpha-amylase level: a pilot study. Front. Hum. Neurosci. 12:202. 10.3389/fnhum.2018.00202 29977196 PMC6021745

[B107] Võ M. L. Conrad M. Kuchinke L. Urton K. Hofmann M. J. Jacobs A. M. (2009). The berlin affective word list reloaded (BAWL-R). Behav. Res. Methods 41, 534–538. 10.3758/BRM.41.2.534 19363195

[B108] Wagner U. Hallschmid M. Rasch B. Born J. (2006). Brief sleep after learning keeps emotional memories alive for years. Biol. Psychiatry 60, 788–790. 10.1016/j.biopsych.2006.03.061 16806090

[B109] Warren C. M. Tona K. D. Ouwerkerk L. Van Paridon J. Poletiek F. van Steenbergen H.. (2019). The neuromodulatory and hormonal effects of transcutaneous vagus nerve stimulation as evidenced by salivary alpha amylase, salivary cortisol, pupil diameter, and the p3 event-related potential. Brain Stimul. 12, 635–642. 10.1016/j.brs.2018.12.224 30591360

[B110] Warren C. M. van den Brink R. L. Nieuwenhuis S. Bosch J. A. (2017). Norepinephrine transporter blocker atomoxetine increases salivary alpha amylase. Psychoneuroendocrinology 78, 233–236. 10.1016/j.psyneuen.2017.01.029 28232237

[B111] Weymar M. Bradley M. M. Hamm A. O. Lang P. J. (2014). Encoding and reinstatement of threat: recognition potentials. Neurobiol. Learn. Mem. 107, 87–92. 10.1016/j.nlm.2013.11.005 24274959 PMC3902191

[B112] Weymar M. Hamm A.O. (2013). Electrophysiological signature of emotional memories, in Hurting Memories and Beneficial Forgetting, eds Linden M. Rutkowski K. (Elsevier Insights), 21–35. 10.1016/B978-0-12-398393-0.00002-X

[B113] Weymar M. Löw A. Hamm A. O. (2011). Emotional memories are resilient to time: evidence from the parietal ERP old/new effect. Hum. Brain Mapp. 32, 632–640. 10.1002/hbm.21051 21391253 PMC6870483

[B114] Weymar M. Löw A. Melzig C. A. Hamm A. O. (2009). Enhanced long-term recollection for emotional pictures: evidence from high-density ERPs. Psychophysiology 46, 1200–1207. 10.1111/j.1469-8986.2009.00869.x 19674397

[B115] Weymar M. Löw A. Schwabe L. Hamm A. O. (2010). Brain dynamics associated with recollective experiences of emotional events. Neuroreport 21, 827–831. 10.1097/WNR.0b013e32833d180a 20625332

[B116] Weymar M. Schwabe L. Löw A. Hamm A. O. (2012). Stress sensitizes the brain: Increased processing of unpleasant pictures after exposure to acute stress. J. Cogn. Neurosci. 24, 1511–1518. 10.1162/jocn_a_00174 22185496

[B117] Weymar M. Ventura-Bort C. Wirkner J. Genheimer H. Wendt J. Hamm A. (2019). Effects of transcutaneous vagus nerve stimulation (TVNS) on selective attention and emotional episodic memory: findings from ERP research, in Psychophysiology, Vol. 56 (Hoboken, NJ: Wiley), S12'12.

[B118] Wixted J. T. Stretch V. (2004). In defense of the signal detection interpretation of remember/know judgments. Psychon. Bull. Rev. 11, 616–641. 10.3758/BF03196616 15581116

[B119] Wright S. P. (1992). Adjusted p-values for simultaneous inference. Biometrics 48, 1005–1013. 10.2307/2532694

[B120] Yakunina N. Kim S. S. Nam E.-C. (2017). Optimization of transcutaneous vagus nerve stimulation using functional MRI. Neuromodul. Technol. Neural Interface 20, 290–300. 10.1111/ner.12541 27898202

[B121] Yonelinas A. P. (2001). Components of episodic memory: the contribution of recollection and familiarity. Philos. Trans. R. Soc. Lond. Ser. B Biol Sci. 356, 1363–1374. 10.1098/rstb.2001.0939 11571028 PMC1088520

[B122] Yonelinas A. P. Aly M. Wang W.-C. Koen J. D. (2010). Recollection and familiarity: examining controversial assumptions and new directions. Hippocampus 20, 1178–1194. 10.1002/hipo.20864 20848606 PMC4251874

